# Proteome and Secretome Analysis Reveals Differential Post-transcriptional Regulation of Toll-like Receptor Responses*[Fn FN2]

**DOI:** 10.1074/mcp.M116.064261

**Published:** 2017-02-24

**Authors:** Marijke Koppenol-Raab, Virginie Sjoelund, Nathan P. Manes, Rachel A. Gottschalk, Bhaskar Dutta, Zachary L. Benet, Iain D. C. Fraser, Aleksandra Nita-Lazar

**Affiliations:** From the ‡Laboratory of Systems Biology, National Institute of Allergy and Infectious Diseases, National Institutes of Health, Bethesda, Maryland, 20892

## Abstract

The innate immune system is the organism's first line of defense against pathogens. Pattern recognition receptors (PRRs) are responsible for sensing the presence of pathogen-associated molecules. The prototypic PRRs, the membrane-bound receptors of the Toll-like receptor (TLR) family, recognize pathogen-associated molecular patterns (PAMPs) and initiate an innate immune response through signaling pathways that depend on the adaptor molecules MyD88 and TRIF. Deciphering the differences in the complex signaling events that lead to pathogen recognition and initiation of the correct response remains challenging. Here we report the discovery of temporal changes in the protein signaling components involved in innate immunity. Using an integrated strategy combining unbiased proteomics, transcriptomics and macrophage stimulations with three different PAMPs, we identified differences in signaling between individual TLRs and revealed specifics of pathway regulation at the protein level.

The innate immune system is essential for host defense, providing a rapid initial reaction to infection or tissue damage and activating adaptive immunity (1). Pathogen recognition receptors (PRRs)^1^ recognize structures conserved between pathogens (pathogen-associated molecular patterns, PAMPs) (2, 3). The Toll-like receptors (TLRs) are a prototypic PRR family (4) of transmembrane proteins predominantly expressed by professional innate immune cells such as macrophages and dendritic cells. TLRs are located on the cell surface and in endosomes, where they recognize diverse microbial molecules and trigger tightly regulated signaling cascades through a complex network of signal transduction proteins. To date, eleven human and thirteen mouse TLRs have been identified, each of which recognizes specific agonists derived from bacteria, fungi or viruses (5, 6). TLRs differ in their expression profile, ligand specificity and signaling; however, they all activate the NF-κB signaling pathway, the most ancient host defense mechanism found in mammals, plants and insects (7). The downstream signaling events initiated by TLR stimulation can be divided into two main pathways based on the adapter recruited by the activated TLR. Upon stimulation, TLR2 and TLR7 recruit the cytoplasmic adapter myeloid differentiation primary response gene 88 (MyD88). TLR4 is the only TLR that signals through two pathways; the MyD88-dependent response that signals from the plasma membrane and results in production of pro-inflammatory cytokines, and the TRIF (TIR-domain-containing adapter-inducing interferon-β)-dependent response that originates from the early endosome and results in type 1 interferon production (8). TLR4 and TLR2 signal from the cell surface in response to bacterial components and can also signal from the endosome (9–11). TLR7 is located exclusively in the late endosome where it signals in response to its natural ligand, single stranded nucleic acids (8).

Many of the proteins that are responsible for the immune response subsequent to TLR activation are secreted (*e.g.* the cytokines TNF-α and IL6) to propagate the inflammatory response in an autocrine or paracrine manner, attracting and/or activating other immune cells (12). Proteins released from macrophages in response to an invading pathogen are detected by neighboring cells and thus play a key role in immune cell communication. These proteins make up a subproteome referred to as the secretome which comprises of proteins released through various mechanisms including classical and nonclassical secretory pathways as well as exosome-mediated secretion and membrane shedding (13, 14). So far, only a handful of proteomic studies of the secretome response to TLR activation have been reported in the literature, and these studies focused on the stimulation of TLR4 with LPS and did not include other TLR ligands. Although some studies were performed in different cell types (15–17), specific studies investigating the secretome response to LPS stimulated macrophages generated sparse data (18–20).

The inflammatory response is counteracted in various ways by actions on NF-κB itself (21) or by the degradation or destabilization of NF-κB target gene transcripts (22). The second set of regulators is provided by the MAPK pathway, and the type 1 interferon response is regulated by the interferon regulatory factors (IRFs) (23). It is essential that the signaling is robust enough for an appropriate immune response yet carefully balanced so that an infection can be eradicated without over-activation, which could lead to pathological reactions, including septic shock—the major cause of mortality in the case of bacterial infection (24, 25), and autoimmunity. Identifying novel factors involved in the propagation and regulation of TLR signaling is necessary to further our understanding of this important immune response area and can provide insight into autoimmune and inflammatory disorders where TLR signaling is implicated, leading to rational design of vaccines and drugs (26).

Many systems biology studies of TLR signaling in macrophages focus on TLR4 as a model system (27, 28) and rely on transcriptional profiling methods. Although extremely informative, such studies cannot account for all of the differences in the responses to various pathogens. For example, they do not provide information on secreted proteins, which play a critical role in intercellular communications, or on intercellular protein and phospho-protein concentrations, which are the essential components of cellular signaling.

Despite extensive studies, many components of TLR network are unknown and therefore systematic, multidisciplinary discovery is needed to determine the impact of cellular components on the pathway activation (29). Also, gene expression changes in many studies poorly correlate with changes at the functional level (30–38). Thus, proteomic studies are needed to achieve an integrated analysis of cellular processes. In the current study, we used mass spectrometry-based proteomic methods to investigate the intracellular (proteome) and extracellular (secretome) responses of murine macrophages to TLR ligands. This approach enables an unbiased profiling of the protein expression changes in response to TLR stimulation that provides a systems-level characterization of TLR signaling. The comparison to the transcriptome revealed a significant number of proteins regulated at the post-transcriptional level, emphasizing the importance of systematic and global studies that reach beyond gene expression profiling.

## EXPERIMENTAL PROCEDURES

### 

#### 

##### Cell Culture and Reagents

The murine macrophage cell line RAW264.7 was obtained from Sigma-Aldrich Co. (St. Louis, MO). Cells were grown in Dulbecco's Modified Eagle Medium (DMEM) supplemented with 2 mm l-glutamine, 20 mm HEPES and 10% fetal bovine serum (FBS) in a humidified incubator at 37 °C, 5% CO_2_ and passaged every 2–3 days on sterile tissue-culture treated plates. DMEM medium, HEPES and l-glutamine were obtained from Lonza (Walkersville, MD), and FBS from Gemini Bio-Products (West Sacramento, CA).

Cell viability was assessed using DEAD green stain (Thermo Fisher Scientific, Waltham, MA) according to manufacturer's protocol upon stimulation of the cells with 100 ng/ml lipopolysaccharide (LPS) from *Salmonella Minnesota* R595 (Enzo Life Sciences Inc., Plymouth Meeting, PA) in serum-containing and serum-free media at 0, 3, and 24 h.

For SILAC labeling, DMEM with stable glutamine deficient in arginine and lysine (Cambridge Isotope Laboratories, Tewksbury, MA) was supplemented with 10% FBS and 20 mm HEPES. Stable isotopes were introduced by adding unlabeled (Sigma-Aldrich Co.) or stable isotope-labeled (Cambridge Isotope Laboratories) l-arginine·HCl and l-lysine·2HCl at concentrations of 0.398 mm and 0.798 mm, respectively. Light media contained unlabeled l-arginine (Arg0) and l-lysine (Lys0), medium media contained ^13^C_6_-l-arginine (Arg6) and ^2^D_4_-l-lysine (Lys4), and heavy media contained ^13^C_6_^15^N_4_-l-arginine (Arg10) and ^13^C_6_^15^N_2_-l-lysine (Lys8). After five passages on tissue culture dishes, incorporation of the isotopes was evaluated in whole cell lysates by mass spectrometry.

##### Preparation of the Proteome Samples

For each labeling condition, RAW264.7 macrophages were grown in 12-well dishes, seeded at a concentration of 10^6^ cells/ml, 1 ml per well, and incubated overnight at 37 °C, 5% CO_2_. The light (Arg0, Lys0)-labeled cells were left untreated, whereas the medium (Arg6, Lys4)-labeled cells were treated for 6 h and the heavy (Arg10, Lys8)-labeled cells were treated for 12 h with either 100 ng/ml lipopolysaccharide (LPS) from *Salmonella Minnesota* R595 (Enzo Life Sciences Inc.), 1 μm resiquimod (R848) (Enzo Life Sciences Inc.), or 1 μm Pam3CSK4 (P3C) (Invivogen, San Diego, CA). A basal state, unstimulated time course of nontreated light, medium, and heavy cells was also performed with lysates collected at 0, 6, and 12 h. Each treatment was performed in quadruplicate. At the designated timepoints post-treatment, the cells were washed three times with ice-cold phosphate-buffered saline and lysed in 50 μl modified RIPA buffer (50 mm Tris pH 7.5, 150 mm NaCl, 1 mm EDTA, 0.1% Na-deoxycholate, 1% IGEPAL) containing protease and phosphatase inhibitors (Roche, Indianapolis, IN). The cell lysate was kept on ice for 20 min with occasional vortexing. Cell lysates were centrifuged at 12,000 *g* at 4 °C for 10 min and the resulting supernatant was collected for proteomic analysis. The protein concentration was determined using the bicinchoninic acid assay (Thermo Fisher Scientific Inc.). The light (untreated), medium (6 h) and heavy (12 h) stimulated cell lysates were combined at a 1:1:1 ratio (w/w), using 10 μg total protein from each time point.

##### Preparation of the Secretome Samples

SILAC-labeled RAW264.7 cells were seeded in 12-well dishes at a concentration of 10^6^ cells/ml, 1 ml per well, and incubated overnight at 37 °C, 5% CO_2_. Prior to treatment with TLR ligands, the media containing FBS was removed, and the cells were washed once with serum-free media. The light (Arg0, Lys0)-labeled cells were left untreated for 24 h in serum-free media. The medium (Arg6, Lys4) and heavy (Arg10, Lys8)-labeled cells were treated for 6 and 24 h, respectively, with either 100 ng/ml LPS, 1 μm R848, or 1 μm P3C in media without serum. A basal state, unstimulated time course was performed in serum-free media with samples collected at 0, 6, and 24h. Each treatment was performed in quadruplicate. At the designated timepoints, 900 μl of conditioned media was removed and filtered using centrifugal filter units with a 0.22 μm pore size (EMD Millipore, Tullagreen, Ireland) to remove any dead cells. The filtered media was flash-frozen in liquid nitrogen and stored at −80 °C. The light (untreated), medium (6 h), and heavy (24 h) stimulated samples were combined at a 1:1:1 ratio (v:v) using 250 μl from each time point. The samples were then concentrated by vacuum centrifugation.

##### Pathogen Challenge

SILAC-labeled RAW264.7 cells were seeded in 48-well plates at a concentration of 2.5 × 10^5^ cells/well, and incubated overnight at 37 °C, 5% CO_2_. Prior to treatment with pathogens, the media containing serum was removed, and the cells were washed once with media lacking serum. The light (Arg0, Lys0)-labeled cells were left untreated for 24 h in serum-free media. The medium (Arg6, Lys4) and heavy (Arg10, Lys8)-labeled cells were treated with pathogens for 6 and 24 h, respectively, in media lacking serum.

*Pseudomonas aeruginosa* was used as an exemplar Gram-negative pathogen signaling through TLR4, *Staphylococcus aureus* as a Gram-positive pathogen signaling through TLR2, and *Burkholderia cenocepacia* as an intracellular pathogen signaling through endosomal TLR7. Cultures of *Pseudomonas aeruginosa* GFP, PAO1 pMRP 9–1 (from Dr. Bradley Borlee, University of Washington, Seattle, WA (39)), *Staphylococcus aureus* FDA209 (ATCC, Manassas, VA), and *Burkholderia cenocepacia* J2315 (a prototypic strain of the highly transmissible ET12 clone (40, 41)) were inoculated in Luria-Bertani medium and grown overnight at 37 °C with shaking. Aliquots of pelleted *P. aeruginosa* and *S. aureus* were washed with normal saline and heat killed at 65 °C and 90 °C, respectively, for one hour. SILAC-labeled RAW264.7 cells were treated with heat-killed *P. aeruginosa* or *S. aureus* at a ratio of 20 bacteria per macrophage, or with live *B. cenocepacia* at a ratio of one bacterium per macrophage. For the *B. cenocepacia* challenge, the plates were centrifuged for 5 min at 200 × *g*, and then incubated at 37 °C in 5% CO_2_. After 1 h of *B. cenocepacia* infection, the treatment medium was removed, and the cells were washed three times with PBS to remove extracellular bacteria and incubated with an antibiotic combination of 250 μg/ml gentamicin (Sigma-Aldrich Co.) and 500 μg/ml of ceftazidime (Sigma-Aldrich Co.) for 2 h to kill the remaining extracellular bacteria (42). After 2 h the media with antibiotics was removed and replaced with serum-free media for the remainder of the treatment time course.

At the designated timepoints, 250 μl of conditioned media was removed and filtered using centrifugal filter units with a 0.22 μm pore size to remove any dead cells. The filtered media was flash-frozen in liquid nitrogen and stored at −80 °C. The light (untreated), medium (6h), and heavy (24 h) stimulated samples were combined at a 1:1:1 ratio (v:v) using 250 μl from each time point. The combined samples were concentrated by vacuum centrifugation.

##### SDS-PAGE separation and in-gel trypsin digestion

The proteome and secretome samples were separated by one-dimensional SDS-PAGE using NuPage 10% or 4–12% Bis-Tris gels with NuPage MES or MOPS running buffer (Life Technologies Corp., Carlsbad, CA). Gels were stained with Colloidal Coomassie blue (SimplyBlue™ SafeStain, Life Technologies Corp.). After destaining, each gel lane was cut into 20 bands and each band was cut into approx. 1 mm^3^ cubes for in-gel trypsin digestion (43). Briefly, the gel cubes were dehydrated with acetonitrile, reduced with 10 mm dithiothreitol in 100 mm ammonium bicarbonate for 30 min at 56 °C. The gel pieces were again dehydrated with acetonitrile and alkylated with 55 mm iodoacetamide in 100 mm ammonium bicarbonate for 20 min at room temperature in the dark. After a third dehydration step with acetonitrile the gel pieces were saturated with 13 ng/μl trypsin in 10 mm ammonium bicarbonate containing 10% (v:v) acetonitrile. Digestion was allowed to proceed overnight at 37 °C. Peptides were extracted from the gel pieces with 1:2 (v:v) 5% formic acid/acetonitrile after incubation for 15 min at 37 °C. The organic solvent was removed from the extracts using a vacuum centrifuge and the dried peptides were resuspended in 0.1% (v:v) formic acid for LC-MS/MS analysis.

##### Peptide Sequencing by Tandem Mass Spectrometry

All LC-MS analyses were performed using an Eksigent nano-LC system (ABI Sciex, Framingham, MA) directly coupled to an LTQ Orbitrap Velos mass spectrometer (Thermo Fisher Scientific Inc., Waltham, MA) that was operated in a data-dependent acquisition mode to automatically switch between Orbitrap full scan MS and LTQ MS/MS using a top 10 method. The single-ligand proteome and secretome samples were run using a 60-min linear gradient. The pathogen-treated secretome samples were run using a 120-min linear gradient and using a precursor ion inclusion list to select specific peptides for fragmentation corresponding to the proteins of interest.

##### Protein Identification and Quantification

Mass spectra were analyzed using MaxQuant version 1.4.1.2 and the Andromeda search engine (44, 45). The maximum mass deviation allowed for the monoisotopic precursor ions was 4.5 ppm for monoisotopic precursors and 0.5 Da for fragment ions. Trypsin was set as the digestion enzyme with a maximum of two allowed missed cleavages. Cysteine carbamidomethylation was set as a fixed modification, and N-terminal acetylation and methionine oxidation were allowed as variable modifications. The spectra were searched using the Andromeda search engine against the mouse Uniprot sequence database (downloaded July 2014, 51574 entries) combined with 247 common contaminants and concatenated with the reversed versions of all sequences. The mass spectrometry proteomics data have been deposited in the ProteomeXchange Consortium via the PRIDE (46) partner repository with the data set identifier PXD004113. Protein identification required at least two unique peptides per protein group. The data were filtered for a 1% FDR at the peptide and protein level. The protein abundance ratios were calculated referring to time 0 h as the reference (the time point choice for proteome and secretome is explained in the above sections on sample preparation). Each time point for each treatment was normalized using the median value to correct for unequal sample mixing. Only the proteins identified by at least two unique peptides and quantified in at least two biological replicates out of the four were considered reliably identified and quantified for use in further analysis. The unstimulated time course samples were used to determine the basal level of variation in protein abundance and secretion in RAW264.7 macrophages over the time course of our experiments (0, 6, and 12 h for the proteome, and 0, 6, and 24 h for the secretome). From these basal fluctuation data we determined fold change thresholds to be considered significant in our TLR-stimulated samples. For the basal proteome, 99% of the proteins did not change their expression levels beyond a fold change of 1.5 during the entire time course of measurement. For the basal secretome, 88% of the proteins exhibited a fold change of less than 2 in their abundance values.

##### Microarray

RAW264.7 cells were treated with individual TLR ligands (100 ng/ml LPS, 1 μm P3C, and 1 μm R848 for stimulation of TLR4, TLR2, and TLR7, respectively) for either 1, 2, or 4 h. Total RNA was isolated from ∼10^6^ cells for each condition with an RNeasy Mini Kit (Qiagen) and high quality RNA was confirmed using a Bioanalyzer 2100 (Agilent Technologies, Columbia, MD). Duplicate biological samples were run for each condition. Amplification and labeling of complementary RNA (cRNA) were performed using the Illumina TotalPrep RNA Amplification Kit (Life Technologies Corp.), and the cRNAs were hybridized to Illumina MouseRef-8 microarrays (Life Technologies Corp.) according to the manufacturer's instructions. The raw intensity values were log_2_-transformed and quantile-normalized for subsequent analyses. The data discussed in this publication have been deposited in NCBI's Gene Expression Omnibus (47) and are accessible through GEO Series accession number GSE85448 (https://www.ncbi.nlm.nih.gov/geo/query/acc.cgi?acc=GSE85448).

##### ELISA

Cytokine output was measured in conditioned media samples by sandwich ELISA in 384-well plates (Thermo Fisher Scientific Inc.) according to the manufacturer's instructions. TNF-α output was measured using the Mouse TNF-α DuoSet ELISA Development System (R&D Systems, Minneapolis, MN). IL6 and IL12p40 output were measured using the respective BD OptEIA mouse ELISA kits (BD Biosciences, San Jose, CA).

##### Data analysis

We used the Database for Annotation, Visualization and Integrated Discovery (DAVID, https://david-d.ncifcrf.gov/; (48, 49)) to assign Gene Ontology (GO) annotations for cellular component (GOCC), molecular function (GOMF), and biological process (GOBP). Obsolete GOCC terms such as “membrane fraction” were ignored for assignment of the main GOCC annotation. Organelle-related GOCC terms were combined into a single GOCC assignment; for example: GOCC assignment “nucleus” used in this study includes the terms nucleoplasm, nuclear membrane, chromosome, chromatin, etc.

Proteins detected in secretome samples were analyzed with the SignalP 4.1 (50) and SecretomeP 2.0 (51) prediction algorithms to determine which proteins are predicted to be secreted via classical (signal peptide-directed) and nonclassical secretion mechanisms.

Principal component analysis (PCA) was performed using the Perseus software version 1.5.0.9 on the 1083 proteins common between the three ligands stimulation data sets for the proteome samples, and the 253 common secretome proteins.

Additional PCA was performed using the MeV software (version 4.8.1) downloaded from http://www.tm4.org/mev.html. For proteome and secretome samples, PCA analysis was carried out separately. For each time point and treatment, median abundance values were calculated across the four biological replicates. Although calculating medians, replicates with missing values were ignored. Samples were projected onto two different two-dimensional planes, consisting of PC1 *versus* PC2 and PC2 *versus* PC3.

##### Experimental Design and Statistical Rationale

For mass spectrometry, all the treatments for the proteome and secretome samples were performed in quadruplicate, starting from the cell culture (biological replicates). There were 12 experimental conditions (proteome and secretome, 2 timepoints, 3 ligands). The proteins were considered if they were found in at least two samples. Untreated cells (time “0”) were used as a control and for the secretome analysis the conditioned media were collected from the cells grown for 24 h in the serum-free media to account for the cell death background.

Hierarchical clustering analyses were performed using Genesis (52). Protein expression fold change values were log transformed (base 2). Samples in this figure were named by appending data type (Sec for secretome and Prot for proteome), treatment (LPS, P3C, and R848), time point (6 h, 12 h, and 24 h), and replicates (letters A–D). Log transformed fold change data showed bell shaped distributions and were symmetric around 0. As the overlap of proteins from secretome and proteome experiments was quite low, only the overlapping proteins from all timepoints and stimulations were used in the clustering analysis. Although the clustering analysis allowed us to directly compare the proteome and secretome data, considerable numbers of proteins, some showing strong up- and downregulation, were not included in the analysis as they were missing in one of the data sets. To specifically focus on proteins showing the strongest changes, we further carried out clustering using proteins showing at least 2-fold up- or downregulation at any of the timepoints and stimulations. For computing fold changes, we used the same sampling nomenclature as above, except for the replicate names as they were averaged. Even if a protein was only present in one data set (proteome or secretome) it was included in the analysis (missing values are colored gray).

Overlap analysis was carried out between the proteome and secretome data from the different stimulations and timepoints. Specifically, the diagonal elements of this matrix show the numbers of proteins identified for each of the experimental conditions. The numbers varied significantly between the different experiments with a general trend that the numbers of proteins from the proteome analyses are always higher than the corresponding secretome analysis. The values in the upper triangle of the matrix are the numbers of proteins overlapping between the different conditions. The lower triangle of the matrix shows the normalized percentage overlap of the proteins between two conditions (calculated by dividing the number of overlapping proteins by the total number of proteins).

##### Correlation of Proteome and Secretome Data with Microarray Data

Protein IDs from the proteome and secretome data were mapped to gene symbols. Pairwise correlations were calculated between log transformed (base 2) proteome/secretome fold change data (three treatments, two timepoints, and two data types) with microarray log transformed fold change data from twelve experimental conditions (untreated and three treatments and three timepoints). Corresponding untreated samples were used for computing fold change in each of the data sets. For computing each pairwise correlation, microarray data were merged with the corresponding proteome/secretome data using gene symbols. For merging two types of data tables we used “inner join,” which means only genes present in both transcriptome and secretome/proteome data tables will be used. Pearson correlation was calculated using the R function “cor.” If multiple measurements were present for a gene symbol, they were averaged.

## RESULTS

### 

#### 

##### Quantitative Proteome and Secretome Analysis of the TLR Responses in Macrophages

**Fig. 1.**
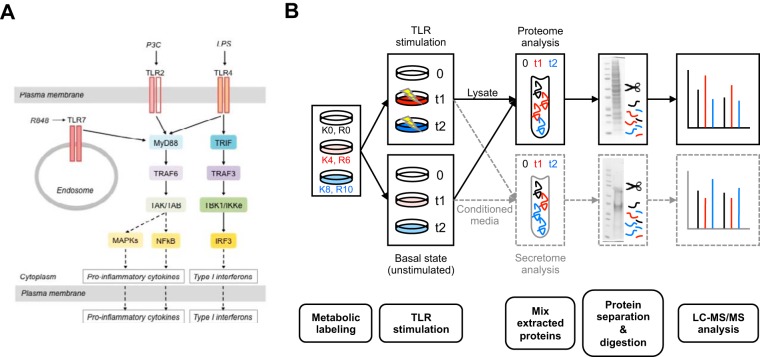
**Experimental design used to identify and quantify changes in the proteome and secretome during macrophage stimulation with TLR ligands.** *A*, A schematic diagram of the canonical Toll-like receptor signaling through TLR2, TLR4 and TL7. *B*, 3-plex SILAC strategy with LC-MS/MS was used to study the effects of TLR stimulation. RAW264.7 cells were labeled with heavy (K8, R10), medium (K4, R6) and light (K0, R0), isotopes in culture and simulated with one of the three TLR ligands (LPS, P3C, or R848) or left unstimulated. The cell lysates were collected for the proteome study and conditioned media were collected for the secretome study. The proteins were extracted from each of the three samples, the samples were combined, the proteins were separated via SDS-PAGE and digested with trypsin as described in Experimental Procedures. Four biological replicates were used to perform independent experiments for each type of analysis.

To examine the signaling pathway activation after different TLR stimulations (Fig. 1*A*), we performed a global and quantitative mass spectrometry-based proteomic analysis of the intracellular protein changes (proteome) and extracellular protein response (secretome) of TLR-stimulated RAW264.7 macrophages using the established strategies of SILAC quantification, SDS-PAGE fractionation, and high-accuracy mass spectrometry (53, 54). The macrophage proteins were SILAC labeled with three distinct isotopic forms of both arginine and lysine to facilitate relative protein quantification between the timepoints (Fig. 1*B*). The cells were passaged five times in SILAC media resulting in a high incorporation level of the isotopes (supplemental Fig. S1*A*). We analyzed the proteome and secretome changes in macrophages stimulated with LPS (TLR4 ligand), P3C (TLR2 ligand), and R848 (TLR7 ligand). Pools of lysates were prepared from unstimulated RAW264.7 cells and from cells stimulated with each individual ligand for 6 and 12 h for the proteome analysis. We chose these timepoints to capture protein changes arising from early-to-intermediate transcriptional regulation events (6 h stimulation) as well as intermediate-to-late changes in transcriptional regulation (12 h stimulation) (55). Pools of conditioned media from nonstimulated macrophages or cells stimulated for 6h or 24h with each individual ligand were combined for the secretome analysis. Our analysis does not differentiate between the various mechanisms the cell may use to release proteins into the outside environment, thus we define the secretome here as encompassing proteins secreted via classical, nonclassical, and exosomal pathways, as well as proteins shed from the cell surface. This definition has been used in other secretome profiling studies as discussed in (13, 14). A later time point (24 h *versus* 12 h) was chosen for the secretome analysis to account for the lag between changes in protein production and protein secretion and to detect late-stage secreted proteins (19). Effective stimulation of the macrophages was confirmed by two independent methods: (1) assessing the increase in MARCKSL1 (MacMARCKS, MRP), a protein known to be induced by LPS treatment (56), by Western blot after treatment of the cells for 6 and 12 h with all three ligands with respect to the untreated cells (supplemental Fig. S1*B*), and (2) by measuring the levels of TNF-α in the conditioned media of untreated and treated cells for the three TLR ligand stimulations after 6 and 24 h (supplemental Fig. S1*C*).

Using high-resolution MS and the MaxQuant proteomics software package for computational analysis we detected relative protein abundances in the lysates and conditioned media of TLR-stimulated macrophages. For each treatment replicate we required a minimum of two unique peptides per protein group for the identification to be considered reliable. We determined the overlap in protein group identifications between treatment replicates to assess the quality of our proteome and secretome data sets and observe that 75–86% of protein groups are identified in two or more replicates depending on the treatment and data set (supplemental Fig. S2).

##### Overview of the Proteome and Secretome Changes

**Fig. 2.**
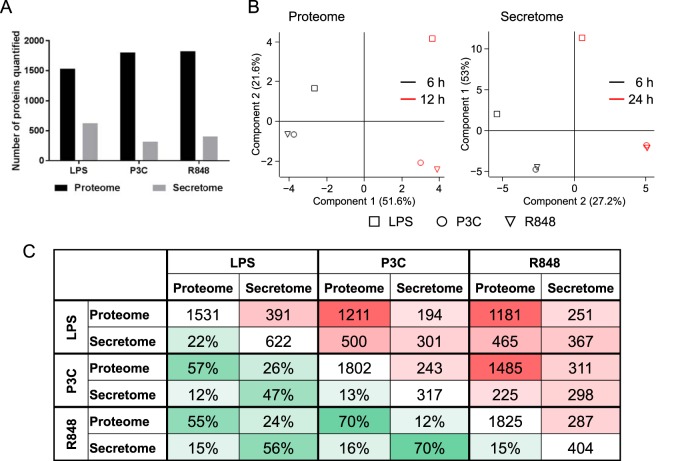
**Global comparison of the proteome and secretome data sets.** *A*, Total numbers of proteins quantified in a minimum of two biological replicates for LPS, P3C, and R848. *B*, Principal Component Analysis with Perseus software. Squares represent the LPS treatment, circles - the P3C treatment, and triangles—the R848 treatment. The 6 h time point is shown in black and the 12 h time point—in red. *C*, Pairwise comparison between the six data sets. The triangle to the right of the diagonal with cells marked in red shows the absolute numbers of identified proteins overlapping between the two given data sets, and the triangle to the left of the diagonal with cells marked in green shows the percentage of proteins common between the two given data sets. The intensity of the color increases with the increase of the overlap.

We investigated the time-dependent changes in protein expression levels following TLR activation by three different ligands. For increased confidence in the protein identification numbers we required that a protein be identified on the basis of at least two unique peptides in a given treatment replicate and data set (proteome or secretome). When assessing the relative protein abundance levels, we considered proteins identified on the basis of at least two unique peptides and quantified in a minimum of two replicates. We identified a total of 1932, 2244, and 2484 proteins across timepoints in lysates of cells stimulated with LPS, P3C, and R848, respectively. Of these, 1531 (LPS), 1802 (P3C), and 1825 (R848) proteins were considered reliably identified and quantified in a minimum of two biological replicates with two unique peptides (Fig. 2*A*). Out of the proteins reliably identified and quantified in each stimulation, 1083 proteins are common to all three ligand stimulations in the proteome data set (supplemental Table S1).

To correlate the intracellular protein response (proteome) of TLR-stimulated macrophages with changes in the extracellular macrophage response following TLR activation, we investigated the changes in the secretome of RAW264.7 cells stimulated for 6 h and 24 h. In total, 947, 506, and 528 proteins were identified for the LPS, P3C, and R848 stimulations, respectively. Of these, we considered 622 (LPS), 317 (P3C), and 404 (R848) secretome proteins to be reliably identified and quantified using the same criteria as for the proteome (Fig. 2*A*). We found 298 proteins common to all three treatments in the secretome data set (supplemental Table S2).

##### Effect of Stress on the Secretome

One of the technical difficulties in measuring the secretome response is that in the absence of stimulation, cells in fresh media will have little to no secreted proteins, making the relative quantification by SILAC challenging. To circumvent this issue, our nontreated samples were collected 24 h after the cells were placed into the fresh media. However, this means that the secretome of our nontreated samples may contain proteins released because of the stress of being cultured for 24 h in serum-free media. To measure this effect, we performed an untreated time course using SILAC-labeled cells switched to serum-free media but not stimulated with TLR ligands. Conditioned media samples were collected at 0, 6, and 24 h, allowing us to compare effects of stress and effects of the stimulation at 24 h (supplemental Fig. S3). It should be noted that for all data presented, only the effect of stimulation is taken into account. The unstimulated time course samples were used to determine the basal level of variation in protein abundance and secretion above which the changes were considered significant (supplemental Fig. S4). Notably, we did not detect increased levels of cell death during the 24 h time course when the cells were grown in the serum-free media (supplemental Fig. S4*B*).

##### Cellular Localization of the Identified Proteins in the Proteome and Secretome

Our analysis detected 993 proteins that were released by macrophages across all ligand stimulations compared with a total of 2951 proteins identified in the macrophage proteome (supplemental Table S3 contains all the results). We used the DAVID bioinformatics tool to assess the subcellular localization of the proteins identified in our proteome and secretome data sets. For the proteome, we observe an enrichment of organelle-associated Gene Ontology cellular compartment (GOCC) terms, with *mitochondrion, nucleus, plasma membrane*, and *endoplasmic reticulum* terms accounting for 57% of the proteins with GOCC assignments (supplemental Fig. S5). In the secretome study, we see the enrichment in the GOCC terms for *extracellular, cytoskeletal*, and large protein complexes including GOCC terms *proteasome, ribosome, ribonucleoprotein complex,* and *translation complex*. We used the SignalP and SecretomeP prediction algorithms to determine which of the proteins in our secretome data set are predicted to be secreted via classical and nonclassical (leaderless) secretion pathways, respectively. Of the proteins detected in the secretome, 183 are predicted to contain a signal peptide, whereas 194 proteins are predicted to be secreted via the nonclassical secretory pathway (supplemental Table S4). Together, these predictions account for ∼38% of the proteins we detect in conditioned media. In addition to classical and nonclassical secretion, proteins can also be released via exosomes, and there is mounting evidence that cells use these vesicles for intercellular communication (discussed in (57)). We investigated if exosome-related proteins were present in our data sets and observe seven of the top 10 exosomal markers listed in ExoCarta (http://exocarta.org/index.html) in our proteome and secretome data sets (CD9, HSPA8, PDCD6IP, ANXA2, SDCBP, ENO1, and HSP90AA1). Interestingly, only one of these, ANXA2, is predicted to be secreted using the SecretomeP prediction algorithm and none are predicted to contain a signal peptide. Thus, these algorithms alone cannot predict which subsets of proteins may be released from cells, and the evolution of the term “secretome” as discussed in (13, 14) reflects the observation that proteins from various subcellular locations may be released by different mechanisms to play a role outside of the cell.

##### The TLR4 Response is Distinct from the TLR2 and TLR7 Responses

Principal component analysis (PCA) of the overall proteome or secretome response to the different ligands shows a clear separation by treatment time but also that the TLR4 (LPS) response is distinct from the TLR2 and TLR7 responses, which show little separation for either time point (Fig. 2*B*). For the proteome and secretome data sets, the first three principal components together captured 80% (PC1: 40%, PC2: 26%, PC3: 14%) and 96% (PC1: 85%, PC2: 7%, PC3: 4%) variability in the data, respectively. As the first three components captured 80% or more variability in the data, we only showed these three components (supplemental Fig. S7). Proteins common to all three stimulations in the proteome data set are well separated with the experimental timeline captured by principal component 1 and stimulation type by principal component 2. PCA of the common secretome shows that the 6 h LPS response is distinctly different from the rest of the secretome samples. Two additional principal component axes are necessary to distinguish secretome timepoints and stimulation type.

Pairwise comparison of the six data sets shows the highest overlap in identified proteins between the TLR2 and TLR7 stimulations with 70% overlap in both the proteome and secretome data sets, weighted to account for the differences in group sizes (Fig. 2*C*). By contrast, the overlap with the TLR4 response is 57 and 55% for the TLR2 and TLR7 proteomes, respectively, and 47 and 56% for the TLR2 and TLR7 secretomes, respectively.

##### Shared Proteins Exhibit Different Temporal Responses

**Fig. 3.**
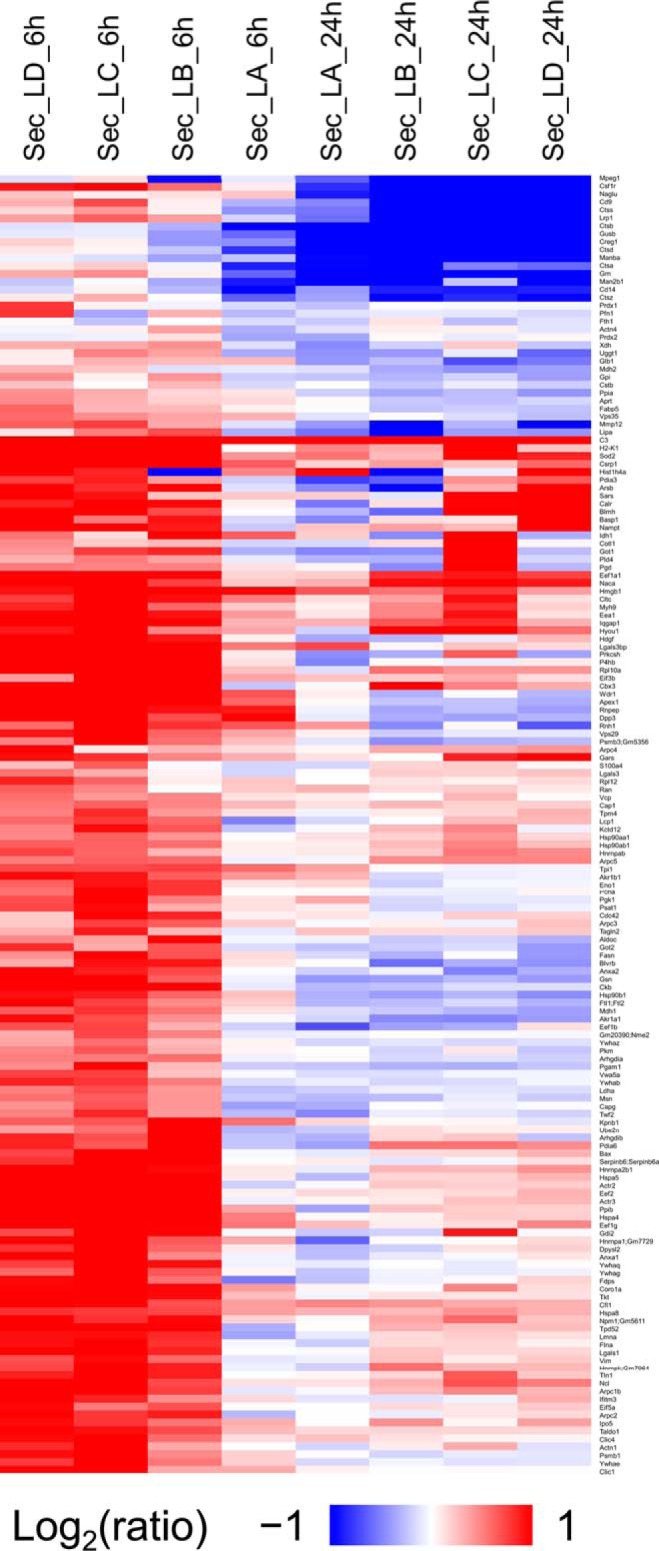
**Changes in the proteins common to all the data sets.** The heatmap represents the hierarchical clustering of the common proteins in the secretome for all the timepoints for the cells treated with LPS. The color key above represents the changes (log2 scale), from dark blue representing the largest decrease, to red representing the largest increase. Cells colored gray represent missing data. Each row is a protein and each column is a sample. Samples are named based on data type, treatment type, time point and replicate as described in “Data analysis”.

Given the overlap in the signaling components used by the different TLRs, we evaluated the responses of the common proteins identified in all three stimulations in both the proteome and the secretome. Hierarchical clustering analysis of these common proteins reveals that this subset of proteins for the secretome behaves like the entire secretome: the LPS stimulation clusters together for the 6 and 24 h stimulation (Fig. 3 depicts LPS stimulated secretome samples) and the P3C and R848 responses cluster together for the 6 h stimulation and the 24 h stimulation, respectively, but overall, the 24 h secretome response to the three stimulations is significantly different from the other timepoints and treatments (supplemental Fig. S6 - 24h secretome data for all the treatments are clustered on the right). At the 6 h time point, TLR2 and TLR7 secretome responses cluster together, whereas the TLR4 response is more separated. On the other hand, this subset of proteins for the proteome has a different clustering pattern than the global proteome response discussed above. The LPS treatment no longer clusters separately from P3C and R848 and the different treatments cluster by time point, with LPS segregating from P3C and R848 within each time point. The general magnitude of up-regulation and down-regulation is also much higher in the secretome data than the proteome data.

##### Most Evident Protein Level Changes in the Proteome and Secretome

**Fig. 4.**
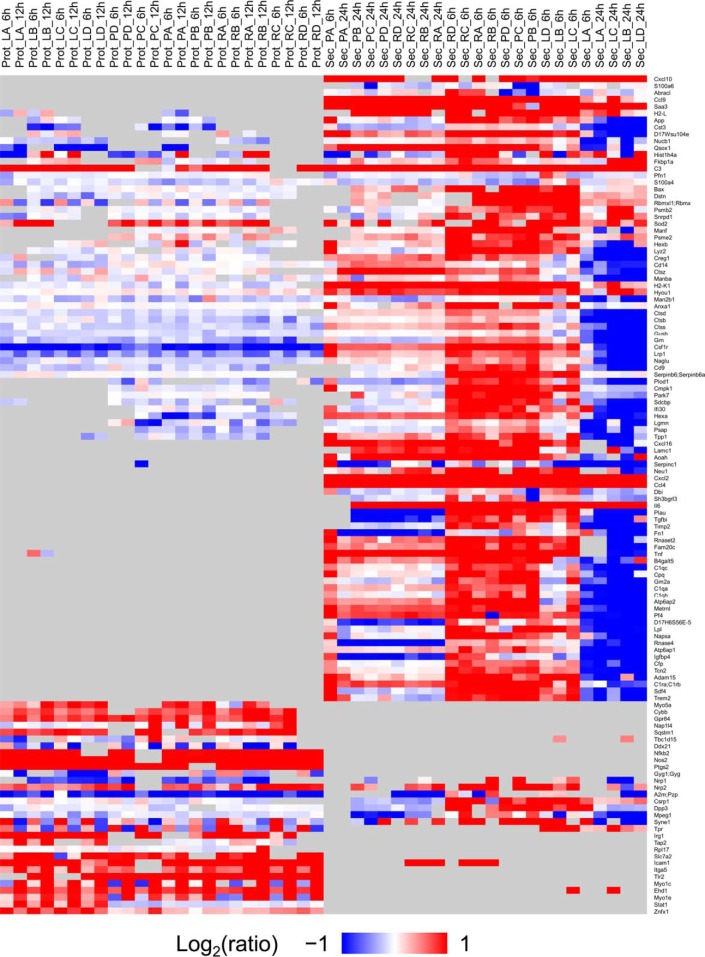
**Heatmap of proteins present in either the secretome or the proteome with fold change equal or larger than 2 induced by any TLR treatment.** The color key is on the left and represents the changes (log2 scale, fold change equal or larger than 2 (in either direction), from dark blue representing the largest decrease, to red representing the largest increase. Cells colored gray represent missing data. Each row is a protein and each column is a sample. Samples are named based on data type, treatment type, and time point as described in Data analysis. Proteins (or rows) are sorted (decreasing order) based on average fold change across all treatments.

We found a subset of proteins whose levels changed 2-fold or more (in any direction) in one or more of the treatment conditions for either the proteome or the secretome (Fig. 4). Clustering of this subset of proteins showing expression changes indicate samples from proteome and secretome are completely separated. We also observed this distinctive response of LPS treatment from PCA analysis (Fig. 2*B*). The proteins in the heatmap were sorted in the decreasing order based on the average expression fold change across all stimulations and timepoints. The top five proteins showing the strongest expression fold changes, *i.e.* Cxcl10, Cxcl2, Saa3, Tnf, and Ccl4, were identified only in the secretome analysis. Using this heatmap, we chose proteins from each group for the follow-up targeted proteomics experiment where cells were challenged with whole pathogens, described below.

##### Proteome, Secretome, and Transcriptome Correlation

**Fig. 5.**
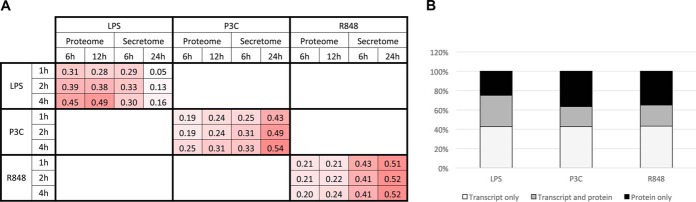
**Correlation between the proteome, secretome and transcriptome.** *A*, Pearson correlation values for the proteome and secretome data with microarray data from RAW264.7 cells treated with the same TLR ligands for one, two, or four hours. Log transformed (base 2) fold change data was used in call cases. *B*, The proportion of transcripts and proteins that exhibit more than 2-fold change in either direction at the protein level, but not at the transcript level (and *vice versa*) for each treatment.

In addition to our analysis of expression changes at the protein level, we also investigated if there was a correlation with microarray data profiling expression changes at the transcript level from RAW264.7 cells treated with the same TLR ligands for one, two, or four hours (Fig. 5*A*). As untreated samples from the corresponding transcriptome, proteome, and secretome samples were used for calculating fold changes, these samples were not included in correlation analysis. We see a strong correlation (Pearson correlation of >0.5) between the transcriptome data and the 24 h secretome data for the TLR2 (P3C) and TLR7 (R848) stimulations, which is consistent with the lag needed for protein production and export. The TLR4 (LPS) secretome shows the weakest correlation with the transcriptome. This may be because of the large amount of down-regulation we see in the LPS secretome at the timepoints we examined. Interestingly, LPS proteome data shows the strongest correlation with the corresponding LPS transcriptome data. However, no such trend was observed for proteome data with R848 and P3C stimulations, as in both cases strong correlation was observed in secretome and transcriptome data. The signaling pathway stimulated by LPS is quite different from the pathways stimulated by R848 and P3C, which might have contributed to this difference in correlation patterns. Given that regulation of protein levels and activity can occur post-transcriptionally, proteome and secretome studies provide additional information about the cellular response to TLR activation that would be missed when looking at transcriptome data alone.

We queried the transcriptomic and proteomic data sets for proteins that show less than 1.5- fold change in expression at the gene level but more than 1.5-fold change in expression at the protein level in the proteome or 2-fold in the secretome to identify proteins that may be subject to posttranscriptional regulation during the immune response to TLR activation. We observed 82, 70, and 72 proteins that exhibit significant changes in expression level at the protein level, but not at the gene level for the LPS, P3C, and R848 stimulations, respectively (Fig. 5*B* and supplemental Table S5). Included in these are immune-related proteins important for TLR signaling, such as complement C3, lysozyme C2, lymphocyte antigen 86, and IL6.

**Fig. 6.**
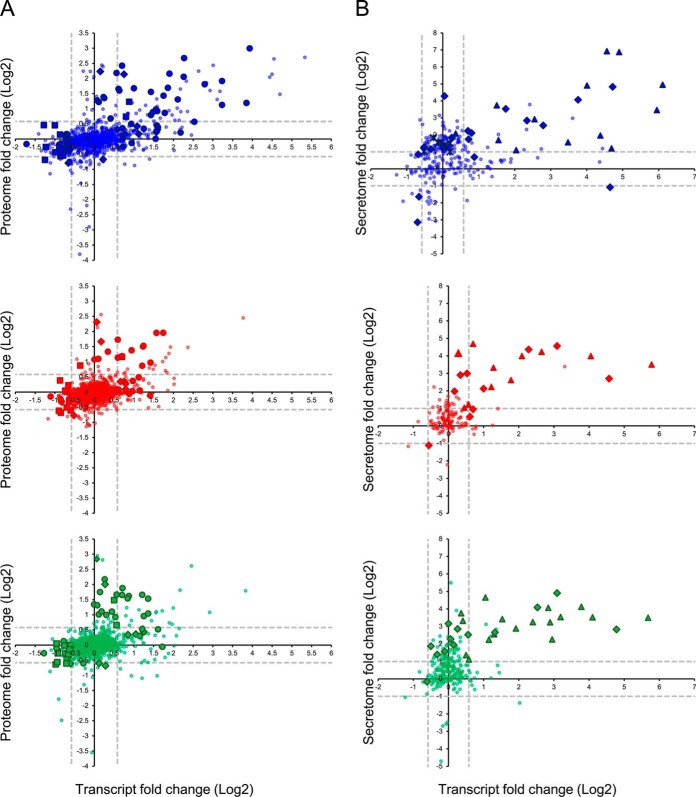
**Enrichment analysis using DAVID.** Three most enriched processes are shown for (*A*) proteome and (*B*) secretome data sets. The color of the symbols indicates treatment type: the blue symbols represent data points from the LPS treatment, the red symbols represent data points from the P3C treatment, and the green symbols represent data points from the R848 treatment. The shape of the symbols indicates the cellular process: circles represent the immune response, diamonds represent the response to wounding, squares represent the DNA metabolic process, triangles represent chemotaxis and crosses represent translation.

We performed the enrichment analysis of the biological processes for the proteins that were up- or down- regulated without a change at the transcript level or *vice versa*, and for the proteins whose changes correlated with the transcript level change. In the proteome data set, the main biological processes identified as enriched by DAVID were: *immune response, response to wounding,* and *DNA metabolic process* (Fig. 6*A*). The *immune response* was controlled both at the transcript and protein level for all three ligands, though the LPS data set had a much stronger transcript component in the response than the P3C and R848 data sets. The *response to wounding* was controlled only at the transcript level for the LPS stimulation (except for NOS2 and PTGS2) whereas both the P3C and R848 stimulations displayed responses that were either solely at the transcript level (for example STAT3, CD44) or at the protein level (for example NOS2, CD81). Both the *immune response* and the *response to wounding* associated genes and proteins were up-regulated for both the transcriptome and the proteome for all three ligands. The *DNA metabolic process* was negatively regulated for all three ligands at the transcript level except for SOD2, which was positively regulated at the protein and transcript level for all three ligands. The *immune response* was ligand dependent with the strongest amplitude in the response seen for the TLR4 stimulation. Both the *response to wounding* and the *DNA metabolic process* did not seem to be ligand dependent. (Fig. 6*A*).

For the secretome (Fig. 6*B*), the three main processes that were enriched are: *translation, chemotaxis* and *response to wounding. Translation* was identified only in the LPS stimulation data set and only at the protein level. *Response to wounding* was identified for the three stimulations at the protein and transcript level. The protein response for all three ligands was mostly positive for all three ligands; the transcript response was mostly positive in the P3C data set whereas the responses to LPS and R848 had more of a negative transcript response component. *Chemotaxis* was also identified for all three stimulations, and the changes were observed at the transcript and protein level. Again, the response amplitude for all three ligands for both the *response to wounding* and *chemotaxis* was not ligand dependent.

##### Whole Bacteria Stimulation Results

**Fig. 7.**
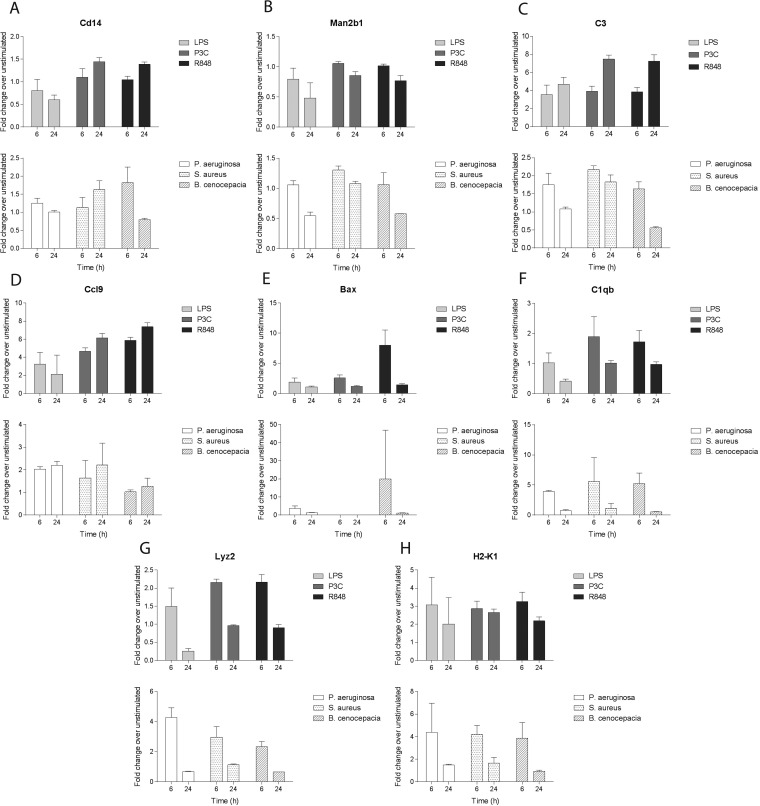
**The targeted proteomics results of the heat-killed pathogen challenge compared with the results obtained for the single ligand stimulations.** Eight representative proteins are depicted. (*A*: Cd14, cluster of differentiation 14; *B*: Man2B1, lysosomal alpha-mannosidase; *C*: C3, complement factor 3; *D*: Ccl9, chemokine (C-C motif) ligand 9; *E*: Bax, Bcl-2-associated X protein; *F*: C1qb, complement factor C1qb; *G*: Lyz2, lysozyme C2; *H*: H2-K1, H-2 class I histocompatibility antigen, K-B alpha chain). The top graphs in each panel indicate fold changes upon 6h and 24h treatments with LPS (*light gray*), P3c (*dark gray*) and R848 (*black*). The bottom panels indicate fold changes upon 6h and 24h treatments with P. aeruginosa (white), S. aureus (dotted) and B. cenocepacia (hatched).

To investigate the changes in protein expression when macrophages are presented with complex combinations of ligands, we performed stimulations of the cells with whole pathogens. A similar time course to the one with the single ligand treatments was performed using heat-killed Gram-positive *S. aureus* or Gram-negative *P. aeruginosa* or live *B. cenocepacia* as an intracellular pathogen. For this analysis, a targeted proteomics approach was used to specifically measure a subset of proteins exhibiting significant fold changes in the secretome samples of the single ligand stimulations. This subset consisted of 28 proteins associated with the top 10 biological process GO terms showing significant regulation (supplemental Table S6). Among these, we chose cytokines (IL6, TNF-α), chemokines (Ccl4, Cxcl2, Ccl9), and complement factors (C1qa, C1qb, C1qc, C3, Cfp), because of their biological importance in the immune response, and other proteins whose fold changes at the protein level were significant. We have obtained quantitative measurements for 24 of the 28 targeted proteins. For most of the targeted proteins we observed the same direction of changes (Fig. 7), although the magnitude of the response varied. The response for some of the proteins suggested roughly the same magnitude of changes when comparing the single-ligand and whole-pathogen stimulations (CD14, Man2b1; Fig. 7*A* and 7*B*). For others, stimulation with whole pathogens suggested a response distinct from the treatment with individual ligands. For example, the results for C3 (Fig. 7*C*) and CCL9 (Fig. 7*D*) suggested a weaker response to whole pathogens than single ligands, whereas other proteins, including Bax, C1qb, Lyz2, and H2-K1 (Fig. 7*E*, 7*F* 7*G*, and 7*H*, respectively), seemed to be more up-regulated with whole pathogen treatment compared with single ligands.

## DISCUSSION

TLRs are essential sensors of the innate immune system and among the first to detect invading microbial pathogens. Each TLR recognizes different microbe-derived molecules and elicits a different immune response despite activating the canonical signaling components that are shared by all TLRs. As a result of these signaling cascades, immune cells release factors important for cellular communication and propagation of the immune response. These proteins can be released by a variety of mechanisms and make up a subproteome known as the secretome. We have used mass spectrometry-based proteomic methods to perform global profiling of both the intracellular and extracellular macrophage responses to three TLR ligands at the protein level to gain a systems-level understanding of this important innate immune signaling network. Of the three ligands used, we observe that the response to TLR4 activation is more distinct than the responses to TLR2 and TLR7 stimulation, which are more similar to each other.

The significant amount of downregulation after the initial burst of upregulation for many inflammatory cytokines during the LPS stimulation may suggest that the TLR4 response, although the most intense, is rapidly shut off. We also observed that the magnitude of upregulation and downregulation is stronger in the secretome data set than the proteome data set. This strong response is likely required to accumulate sufficient signaling components in the extracellular milieu for activation of neighboring cells and propagation of the immune response. The difference between TLR4 and TLR2/TLR7 induced signals can likely be explained by TLR4 uniquely acting through two adaptor pathways, MyD88 and TRIF.

There is continuing debate on the concordance of transcripts and protein abundances (58), and the precise mechanisms that act at the post-transcriptional level remain to be elucidated (59–61). Cross-species comparisons suggest that orthologous protein levels correlate better than the corresponding transcript abundances indicating that the mechanisms to achieve a particular protein level evolve rapidly (35) and may include many different mechanisms such as altered protein stability, translational efficiency and ribosomal abundance (62–64). This could explain why the proteins involved in the innate immune response do not correlate well with their corresponding transcripts as they are the first line of defense and have evolved to act rapidly in the presence of a pathogen. On the other hand, the adaptive immune system needs more time to react and perhaps this can account for a better correlation between the transcript and the protein levels for that particular pathway. The poorer correlation between transcript and protein for the secretome for all three biological processes can be explained in the same manner as above; they are all essential for the survival of the cell and need to be established quickly. We have observed select proteins involved in chemotaxis and the innate immune response that do not display any change in the transcript levels at the timepoints we have investigated (for example, CXCL16, C3, and IL6). However, in a general analysis, chemotaxis proteins exhibit a correlation between transcript and protein in the secretome study. The fact that there are a few proteins that are also involved in chemotaxis and do not show a change in transcript level in our study could be because of a sampling time problem because those proteins are not found in the proteome analysis and thus most likely are not stored in the cell. Accumulation of protein in the media cannot account for the poor correlation because for all three stimulations, proteins belonging to the 20S and the 26S proteasome subunits have been identified in the secretome. Previous studies have shown that the proteasome retains its enzymatic activity in the extracellular environment (65), and this could have contributed to the decrease in the amounts of certain proteins in the conditioned media with time (IL6, TNF-α) even without a change at the mRNA levels (for IL6) or with an increase in mRNA (for TNF-α) at the timepoints included in our study. It is interesting that the immune response processes detected at both the protein level and the protein/transcript level were more robust for the LPS stimulation than for the other two ligands. Perhaps the enrichment is because of the dual (MyD88/TRIF) signaling pathway of TLR4, and the late signaling component changes of LPS are captured at the transcript/protein level, but the changes because of the early signaling are not captured at the transcript level at the timepoints we examined; this needs to be investigated further.

It is challenging to compare our data with previously published data sets (which only report LPS stimulation) because of the differences in experimental parameters, including cell types, cell lines, timepoints, and LPS concentrations. In the most similar analysis by Meissner *et al.*, 2013 (19) (using 200 ng/ml LPS, mouse bone marrow derived macrophage cells, and a 16 h late time point) the general trend for LPS appears similar (see supplemental Fig. S6 of that paper): several cytokines and chemokines, for example TNF-α, increase secretion up to 8 h post-treatment and then decrease. In the current study, our major focus is on the differences between specific TLR ligands, which have not been examined before at the proteome level, and the differences between gene expression and protein levels.

The general agreement in the trend of response to the whole heat-killed pathogens with the purified ligand experiments (66) suggests that the omics studies that use single ligands can, in general, inform about the TLR-induced response to the pathogens presenting these ligands as their dominant PAMPs. The differences in the magnitude of the response may be because of the differences in the concentrations or availability of the single ligands compared with the whole pathogens. Higher than physiological concentrations of single ligands used in our study can explain the weaker responses to the whole bacteria. In contrast, the stronger up-regulation of proteins such as Bax, C1qb, and Lyz2 may indicate that under certain circumstances treatment with complex combinations of ligands can lead to synergistic responses. In other cases, the choice of pathogen may influence differences between soluble ligand and bacterial stimulation results. For example, we could not detect IL-6, Ccl4 and Cxcl2 in the whole pathogen experiment, likely because of the fact that we used *P. aeruginosa* as a Gram-negative pathogen and its LPS does not trigger the secretion of certain cytokines as efficiently as the LPS from *E. coli* or *S. minnesota*. The pro-apoptotic factor Bax showed the strongest up-regulation in response to the intracellular pathogen *B. cenocepacia* (and to R848, the ligand presented by the intracellular pathogens) This is consistent with the induction of cell death as part of the host response to intracellular infection and highlights stimulus specific protein induction detected in our proteomic data.

Our study demonstrates the utility of combining targeted and global proteomic analyses in the study of the innate immune response to invading pathogens. Our findings reinforce the argument that proteomics-related studies complement gene expression studies to show different levels and modes of post-transcriptional regulation (29). Furthermore, as proteomic approaches can explain rapid functional changes supporting effective defense against pathogen, they represent an important direction for the future of systems immunology.

## DATA AVAILABILITY

The mass spectrometry proteomics data have been deposited to the ProteomeXchange Consortium via the PRIDE (46) partner repository with the data set identifier PXD004113. The microarray data have been deposited in NCBI's Gene Expression Omnibus (47) and are accessible through GEO Series accession number GSE85448 (https://www.ncbi.nlm.nih.gov/geo/query/acc.cgi?acc=GSE85448).

## Supplementary Material

Supplemental Data

## Footnotes

^1^ The abbreviations used are:

PRR: pattern recognition receptors
PAMP: pathogen-associated molecular patterns
TLR: Toll-like receptor
NF-κB: nuclear factor kappa-light-chain-enhancer of activated B cells
TNFα: Tumor necrosis factor alpha
IL6: interleukin-6
IL12: interleukin-12.
