# Supplementary material for: Proteome and Secretome Analysis Reveals Differential Post-transcriptional Regulation of Toll-like Receptor Responses
Source: Mol Cell Proteomics. 2017 Feb 24;16(4 Suppl 1):S172–86. doi: 10.1074/mcp.M116.064261 (PMC5393387; doi:10.1074/mcp.M116.064261)
Supplement: Supplemental Data [file supp_16_4-suppl-1_S172__index.html]

Proteome and secretome analysis reveals differential post-transcriptional regulation of Toll-like receptor responses — Proteome and Secretome Analysis Reveals Differential Post-transcriptional Regulation of Toll-like Receptor Responses — Proteome and Secretome Profiling of TLR Stimulation — Supplemental Data 

# Proteome and Secretome Analysis Reveals Differential Post-transcriptional Regulation of Toll-like Receptor Responses

## Supplemental Data

- Table S1 (.xlsx, 549 KB) - Table S1
- Table S2 (.xlsx, 219 KB) - Table S2
- Table S3 (.xlsx, 5.8 MB) - Table S3
- Table S4 (.xlsx, 29 KB) - Table S4
- Table S5 (.xlsx, 102 KB) - Table S5
- Table S6 (.xlsx, 81 KB) - Table S6
- Supplemental Figures and Legends (.pdf, 2.7 MB) - Supplemental Figures and Legends
