# Supplementary material for: Proteome and Secretome Analysis Reveals Differential Post-transcriptional Regulation of Toll-like Receptor Responses
Source: Mol Cell Proteomics. 2017 Feb 24;16(4 Suppl 1):S172–86. doi: 10.1074/mcp.M116.064261 (PMC5393387; doi:10.1074/mcp.M116.064261)
Supplement: Supplemental Data [file 10.1074_M116.064261_mcp.M116.064261-7.pdf]

Supplemental data legends

Figure S1. (A) A representative labeling efficiency example of a peptide containing a lysine residue. Red line indicates position of the unlabeled peptide. (B) Confirmation of the effective stimulation of the cells for the proteome by Western blot (right) and the Western Blot results (semi-quantitative densitometry) compared to the quantitative mass spectrometry results for each ligand at both timepoints using MARCKSL1 as a representative protein. (C) Confirmation of the effective stimulation of the cells for the secretome by the measurement of TNF levels in the conditioned media. The statistical analysis was performed using a two-way ANOVA, and the resulting p-value was less than 0.0001. The horizontal bars indicate statistically significant differences resulting from post-hoc multiple comparisons (significance level = 0.05).

Figure S2. Results overview for the proteome and secretome analysis. Overlap between the four replicates of the proteome (top) and secretome (bottom) analyses of cells stimulated with (from left to right) LPS, P3C and R848.

Figure S3. (A) Determination of the stress effect on the cells after culture in the serum-free media. (B). The visual timeline of the controls versus treated samples.

Figure S4. (A) Determination of the significant fold change thresholds. (B) Assessment of cell death in the serum-supplemented (left) and serum-free (right) media without (black and gray) and with (dark and light blue) LPS stimulation.

Figure S5. Gene Ontology Cellular Compartment assignments of the proteins found in the proteome (black bars) and secretome (gray bars).

Figure S6. Changes in the proteins common to all the datasets. The heatmap represents the hierarchical clustering of the common proteins in the proteome and secretome for all the treatments and timepoints. The color key below represents the changes (log2 scale), from dark blue representing the largest decrease, to red representing the largest increase. Cells colored gray represent missing data. Each row is a protein and each column is a sample. Samples are named based on data type, treatment type, timepoint and replicate as described in “Data analysis”.

Figure S7. Principal component analysis performed using the MeV software. For proteome (A and B) and secretome (C and D) samples, PCA analysis was carried out separately. Samples were projected onto two different two-dimensional planes, consisting of PC1 vs. PC2 (A and C) and PC2 vs. PC3 (B and D).

Table S1. Proteins identified and quantified in each stimulation in the proteome dataset.

Table S2. Proteins identified and quantified in each stimulation in the secretome dataset.

Table S3. Proteins identified in all of the analyses.

Table S4. Proteins identified as containing a signal peptide by SignalP software (Sheet 1) and proteins predicted to be secreted via the non-classical secretory pathway by SecretomeP software (Sheet 2).

Table S5. Proteins that exhibit significant changes in expression level at the protein level, but not at the transcript level for the LPS (Sheet 1), P3C (Sheet 2), and R848 (Sheet 3) stimulations.

Table S6. List of proteins used for the targeted proteomic analysis after the stimulation of cells with heat-killed whole pathogens.

A

Sequence: TEDPSDSGGLGSTADAVGSSLK, K22-Label:13C(6)15N(2) (8.01420 Da)
Charge: +2, Monoisotopic m/z: 1029.99109 Da (+9.75 mmu/+9.46 ppm), MH+: 2058.97490 Da, RT: 28.13 min,

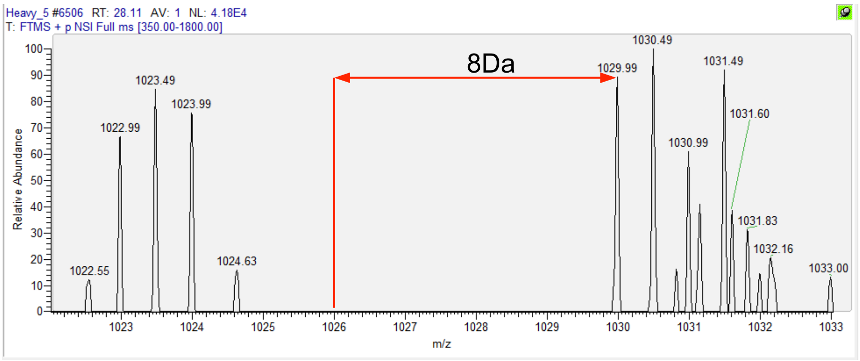

B

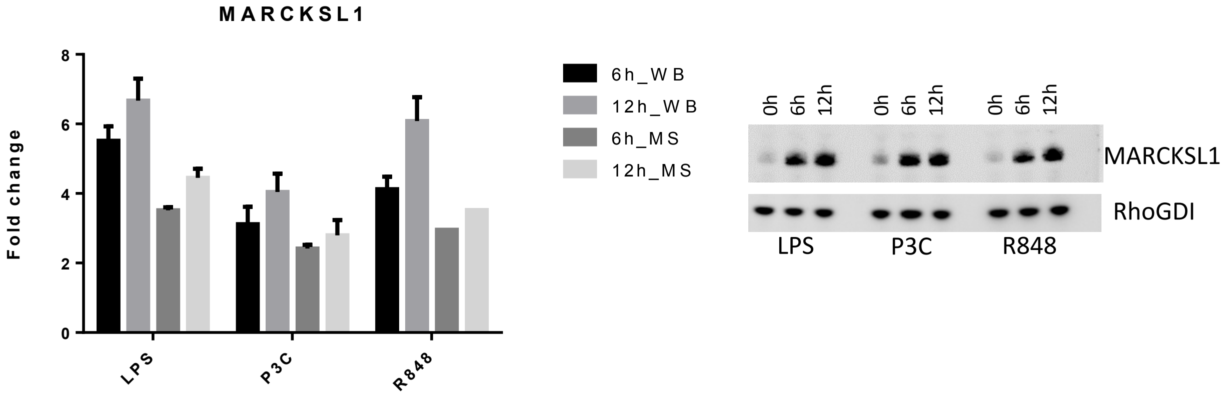

C

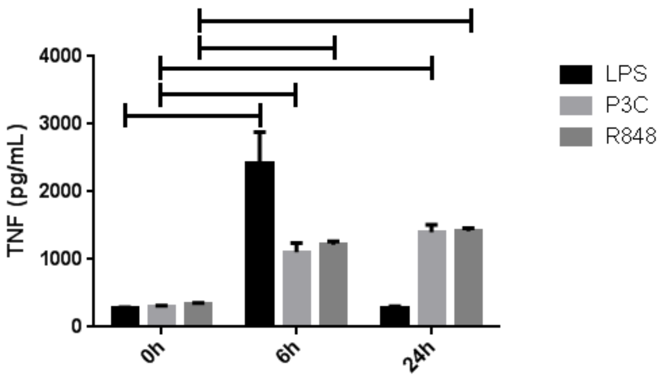

Figure S1

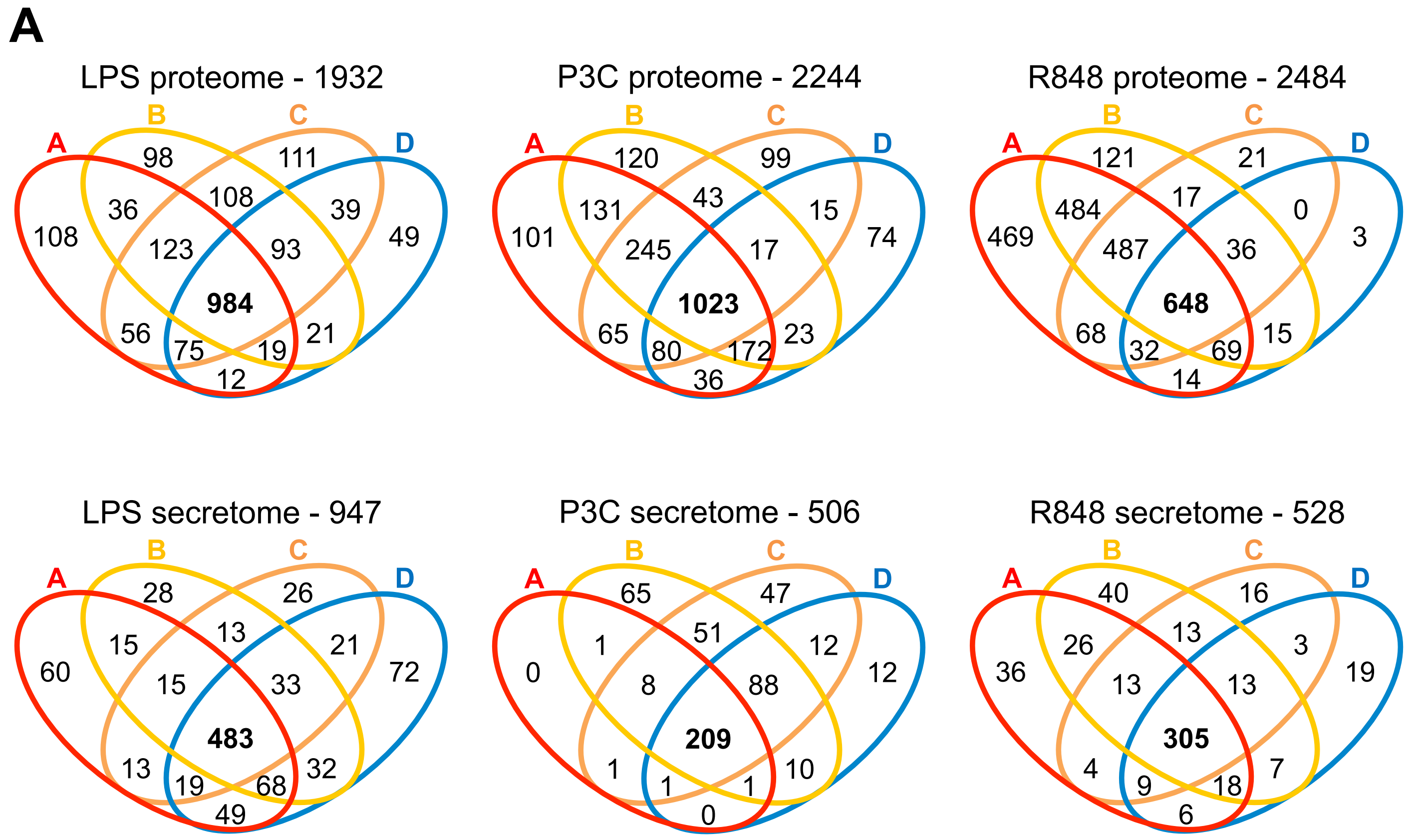
A
LPS proteome - 1932
A B C D
98 111 36 108 39 108 123 93 49 984 56 75 19 21 12
P3C proteome - 2244
A B C D
120 99 131 43 15 101 245 17 74 1023 65 80 172 23 36
R848 proteome - 2484
A B C D
121 21 484 17 0 469 487 36 3 648 68 32 69 15 14
LPS secretome - 947
A B C D
28 26 15 13 21 60 15 33 72 483 13 19 68 32 49
P3C secretome - 506
A B C D
65 47 1 51 12 0 8 88 12 209 1 1 1 10 0
R848 secretome - 528
A B C D
40 16 26 13 3 36 13 13 19 305 4 9 18 7 6

Figure S2

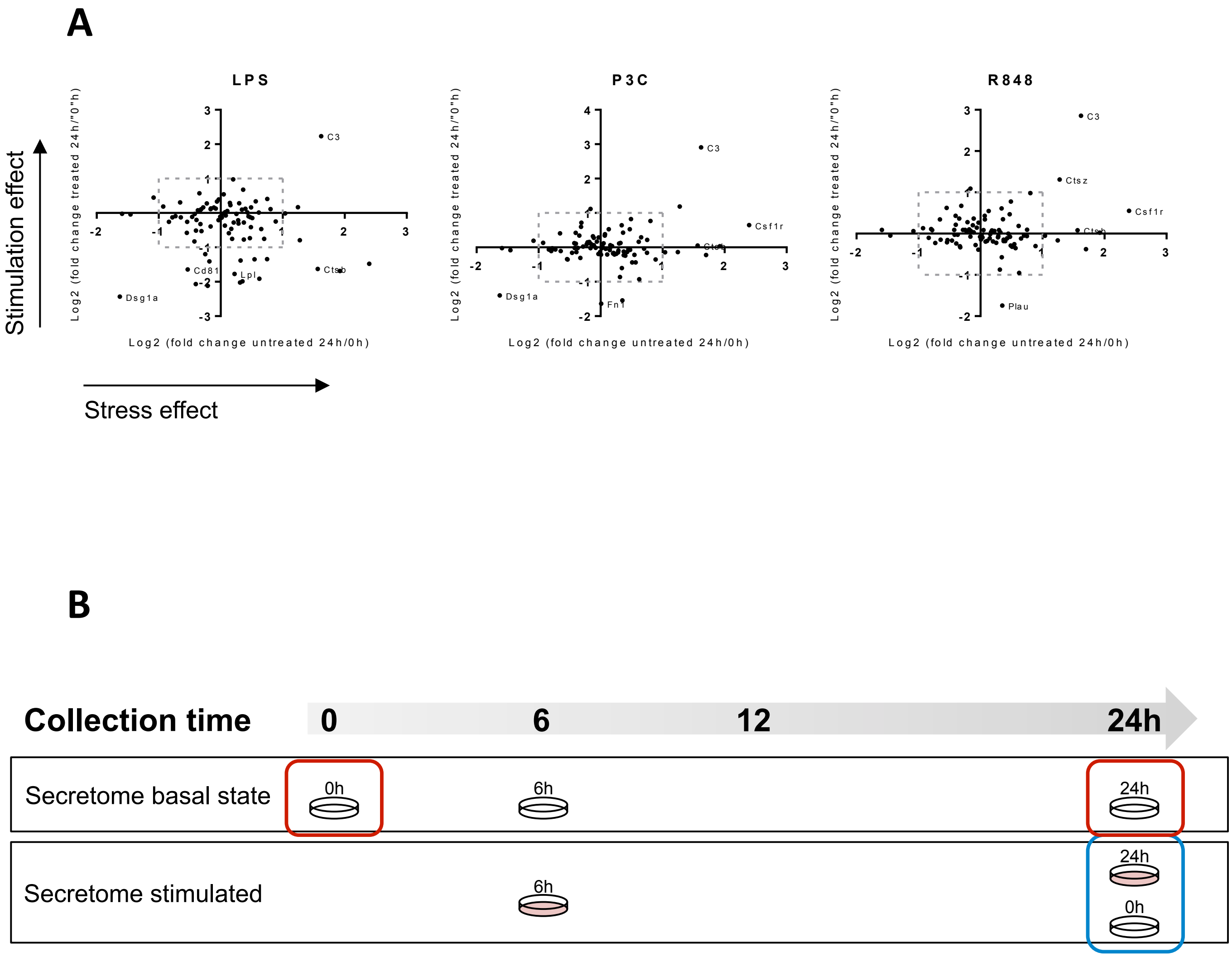
A
LPS
P3C
R848
Log2 (fold change treated 24h/"0"h)
Log2 (fold change untreated 24h/0h)
C3
Cd81
Lpl
Ctsb
Dsg1a
Csf1r
Fn1
Ctsz
Plau
Stimulation effect
Stress effect
B
Collection time
0
6
12
24h
Secretome basal state
Secretome stimulated
0h
6h
24h

Figure S3

A

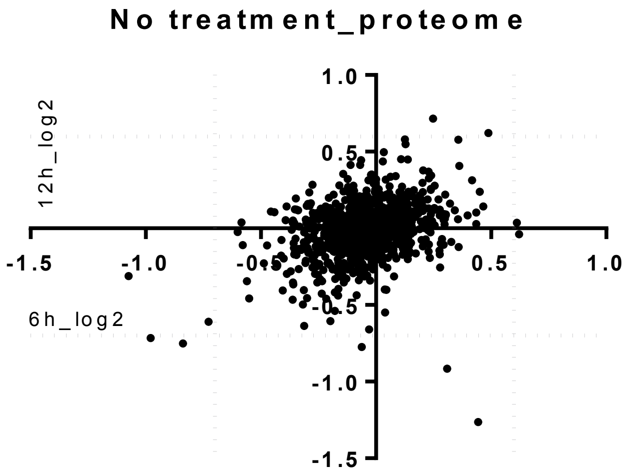

Fold change 1.5
99% proteins within boundaries in time course

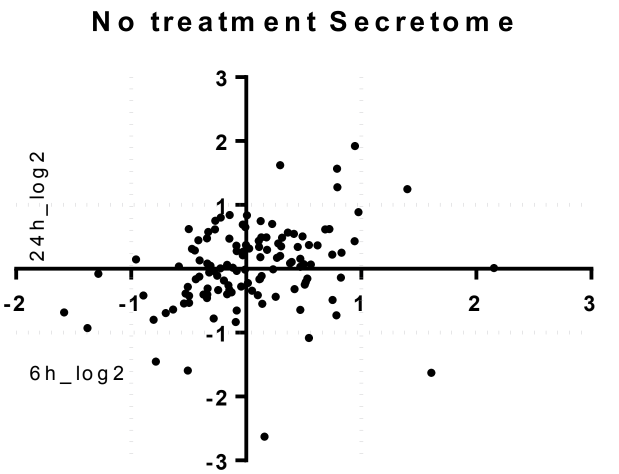

Fold change 2
88% proteins within boundaries in time course

B

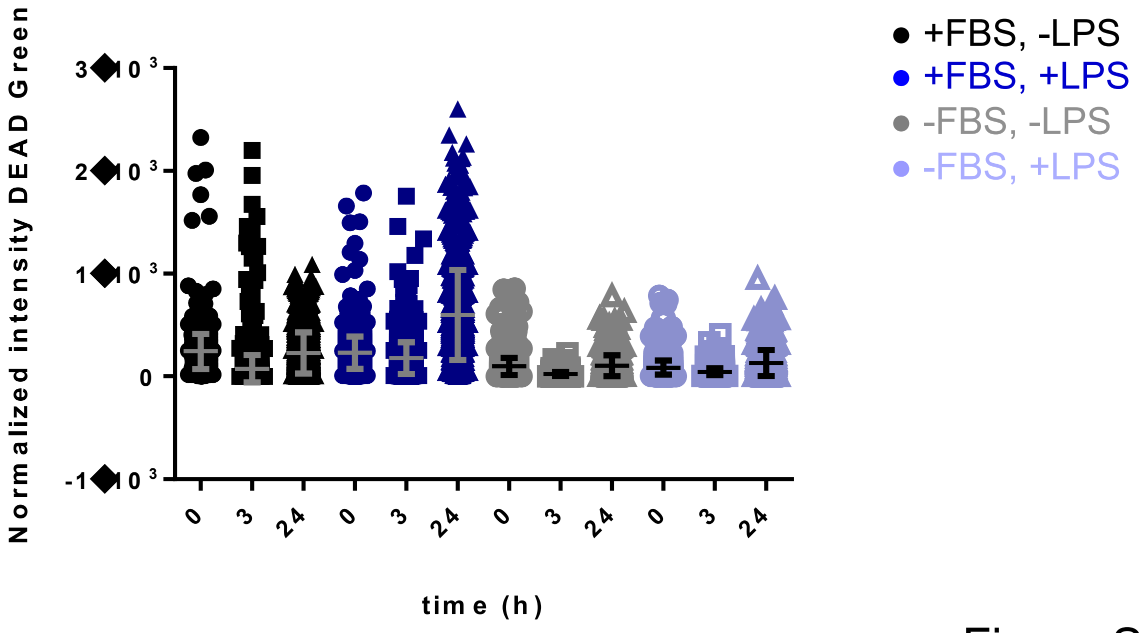

Figure S4

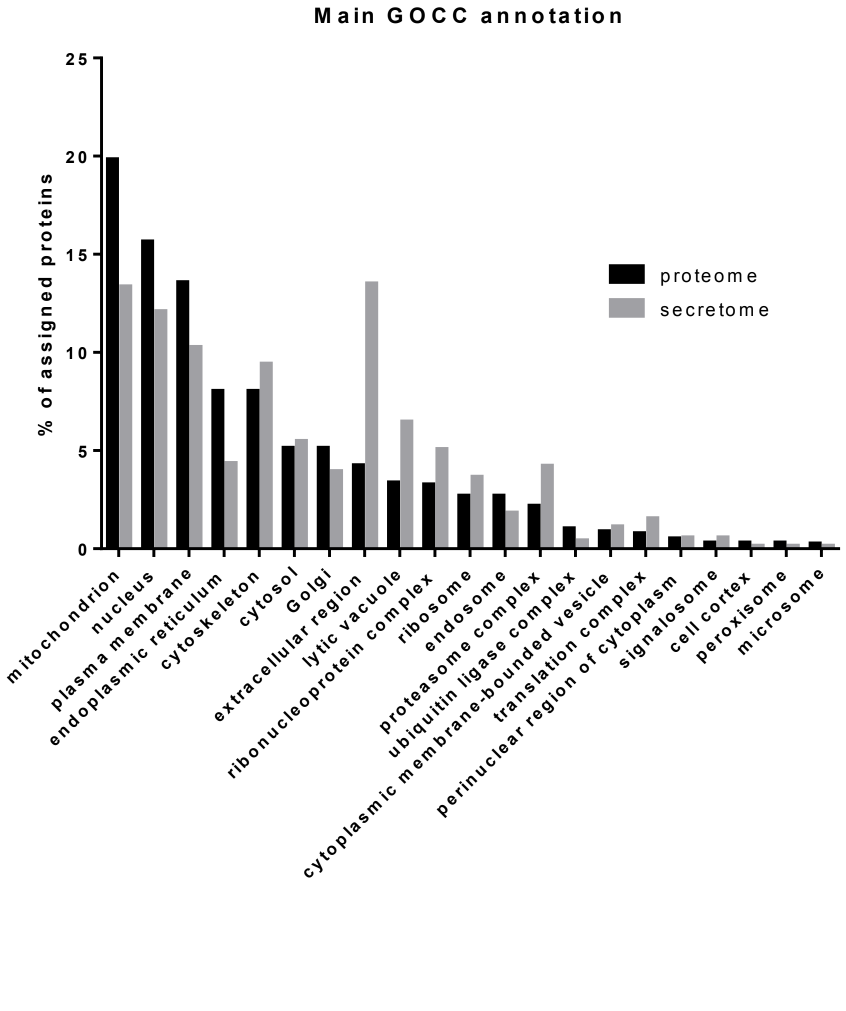

Main GOCC annotation
25
20
15
10
5
0
% of assigned proteins
proteome
secretome
mitochondrion
nucleus
plasma membrane
endoplasmic reticulum
cytoskeleton
cytosol
Golgi
extracellular region
lytic vacuole
ribonucleoprotein complex
ribosome
endosome
proteasome complex
ubiquitin ligase complex
cytoplasmic membrane-bounded vesicle
translation complex
perinuclear region of cytoplasm
signalosome
cell cortex
peroxisome
microsome

Figure S5

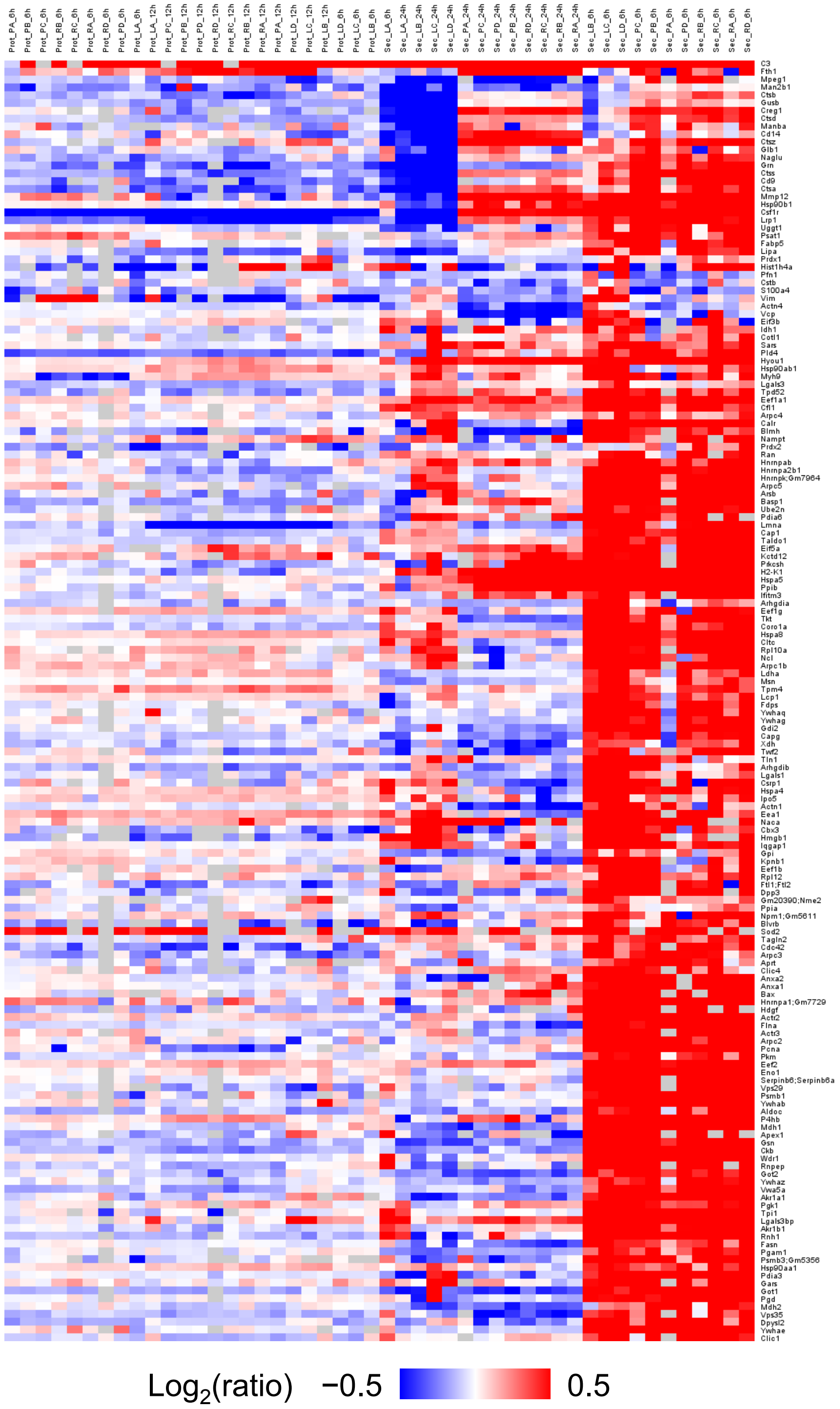

Figure S6

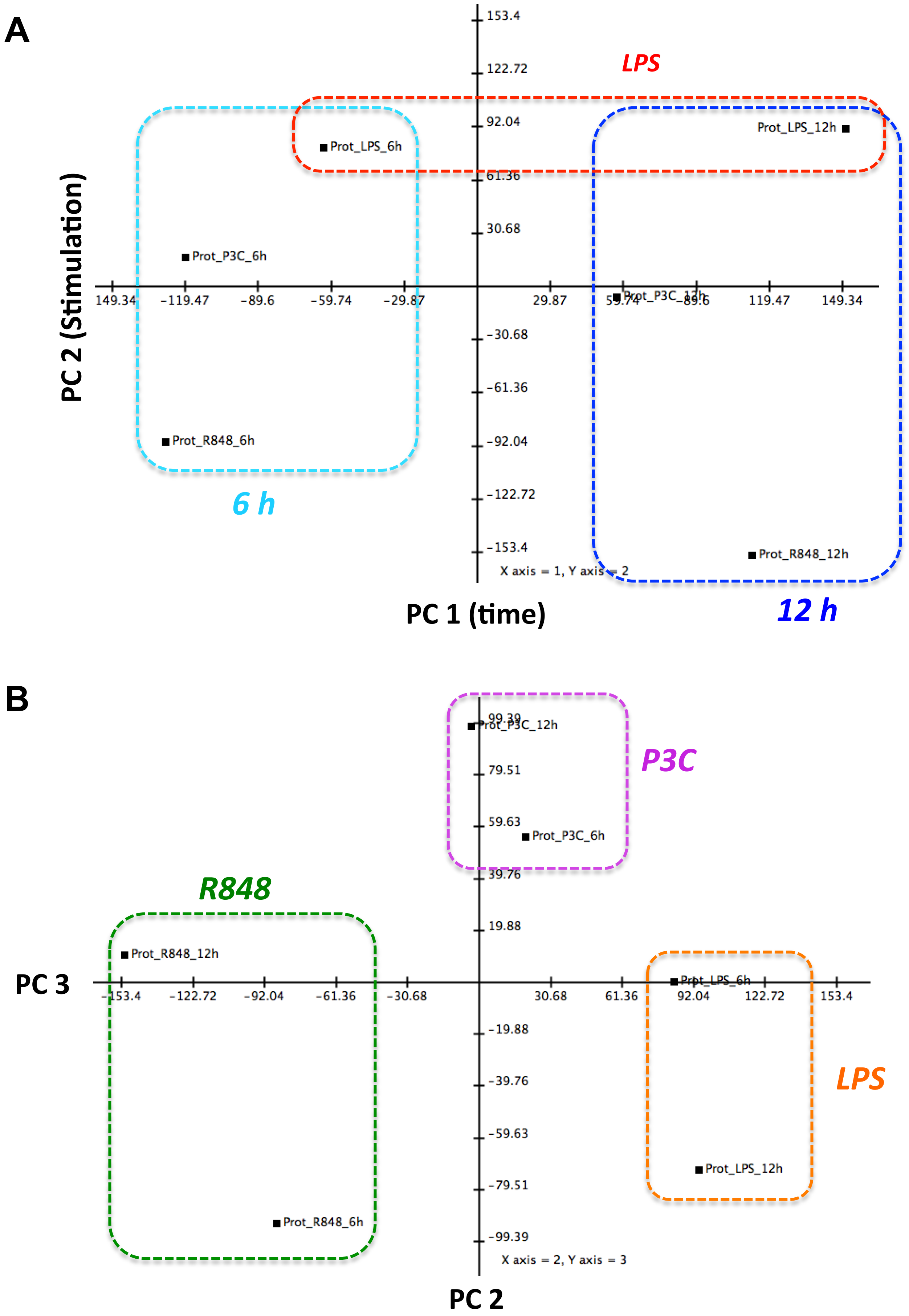

A
153.4
122.72
92.04
61.36
30.68
-30.68
-61.36
-92.04
-122.72
-153.4
149.34
-119.47
-89.6
-59.74
-29.87
29.87
59.74
89.6
119.47
149.34
LPS
Prot_LPS_6h
Prot_LPS_12h
Prot_P3C_6h
Prot_P3C_12h
Prot_R848_6h
Prot_R848_12h
6 h
12 h
X axis = 1, Y axis = 2
PC 2 (Stimulation)
PC 1 (time)
B
99.39
79.51
59.63
39.76
19.88
-19.88
-39.76
-59.63
-79.51
-99.39
-153.4
-122.72
-92.04
-61.36
-30.68
30.68
61.36
92.04
122.72
153.4
P3C
Prot_P3C_12h
Prot_P3C_6h
R848
Prot_R848_12h
Prot_R848_6h
LPS
Prot_LPS_6h
Prot_LPS_12h
X axis = 2, Y axis = 3
PC 3
PC 2

Figure S7

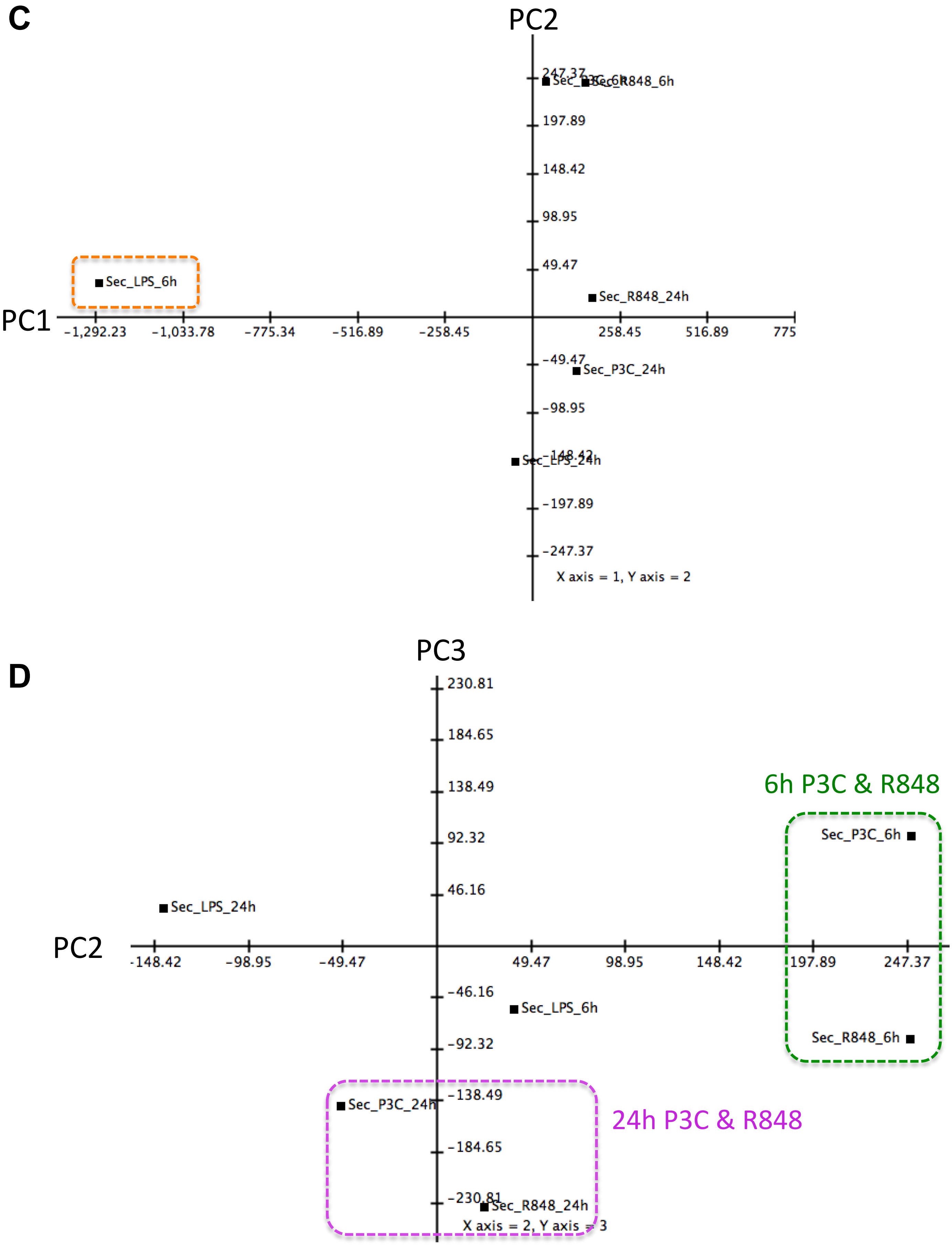
C
PC2
PC1
Sec_LPS_6h
Sec_R848_24h
Sec_P3C_24h
Sec_R848_6h
197.89
148.42
98.95
49.47
-49.47
-98.95
-197.89
-247.37
-1,292.23
-1,033.78
-775.34
-516.89
-258.45
258.45
516.89
775
X axis = 1, Y axis = 2
D
PC3
PC2
230.81
184.65
138.49
92.32
46.16
-46.16
-92.32
-138.49
-184.65
-148.42
-98.95
-49.47
49.47
98.95
148.42
197.89
247.37
6h P3C & R848
Sec_P3C_6h
Sec_R848_6h
Sec_LPS_24h
Sec_LPS_6h
Sec_P3C_24h
Sec_R848_24h
24h P3C & R848
X axis = 2, Y axis = 3

Figure S7
